# Supplementary material for: Understanding the origin of Paris Agreement emission uncertainties
Source: Nat Commun. 2017 Jun 6;8:15748. doi: 10.1038/ncomms15748 (PMC5467211; doi:10.1038/ncomms15748)
Supplement: Supplementary Information — Supplementary Figures, Supplementary Tables, Supplementary Notes and Supplementary References [file ncomms15748-s1.pdf]

Supplementary Note 1: Extrapolation of NDCs defined for 2025

We test the sensitivity of our results to the treatment of NDC targets which do not coincide with the decadal time step of our model. In our default case, we take a conservative approach and assign such NDC targets to the nearest time step. However, in a sensitivity case, we derive custom targets for 2030 for both Brazil and the US and determine their influence on overall emissions. For the US, we assume 2030 emissions reductions as a linear interpolation between their 28% reduction from 2005 values for the year 2025, and their aspirational mid-century target of 83% from 2005 for the year 2050. For Brazil, we construct a sensitivity case which continues the linear reductions implied by the NDC between 2005 and 2025, through to 2030. These updated NDC targets are more ambitious than what could be achieved in SSP3 under default assumptions.

These updated targets result in GHG emissions reductions of about 0.8-1.0 and 0-0.3 $GtCO_2e\ yr^{-1}$ in 2030 in the NAM and LAM regions, respectively. However, due to macro-economic and whole-system-interactions, like global resource prices being lowered, the more stringent emissions reductions in NAM and LAM do not always lead to lower emissions globally. As a result of the implementation of this sensitivity case with two more-stringent regional targets, global emissions vary by -1.2 $GtCO_2e\ yr^{-1}$ to +0.5 $GtCO_2e\ yr^{-1}$. This highlights the importance of considering NDCs in their wider international context.

Supplementary Note 2: Representation of single country NDCs

This study focusses on understanding how large the uncertainties in projected emissions on the global and regional scale under the current formulation of NDCs are, and on the key drivers underlying this uncertainty. There is always a trade-off between the detail of representation of national policies and global and regional feedbacks. For our research question, it is important that global and regional feedbacks are well-represented by the applied modelling framework. The IIASA IAM provides us with such a framework. At the same time, we want to understand how single NDCs are represented and quantified by our framework. We carried out a dedicated sensitivity analysis in which, for one interpretation of the six uncertainty dimensions, we incrementally add single NDCs to their respective region. Combining these estimates with the uncertainties in regional emissions, allows us to understand how single NDCs quantifications with our framework compare to estimates available in the literature. Other modelling frameworks exist, with different regional aggregation (for example, see: http://www.fp7-advance.eu/content/model-documentation). Particularly in regions with many diverse countries of relatively similar size, like sub-Saharan Africa or Latin America, quantifying NDCs at a finer level might show different results. However, at the same time, the fine resolution enables us to compute the necessary large number of scenario variations and to account for the inclusion of macro-economic linkages.

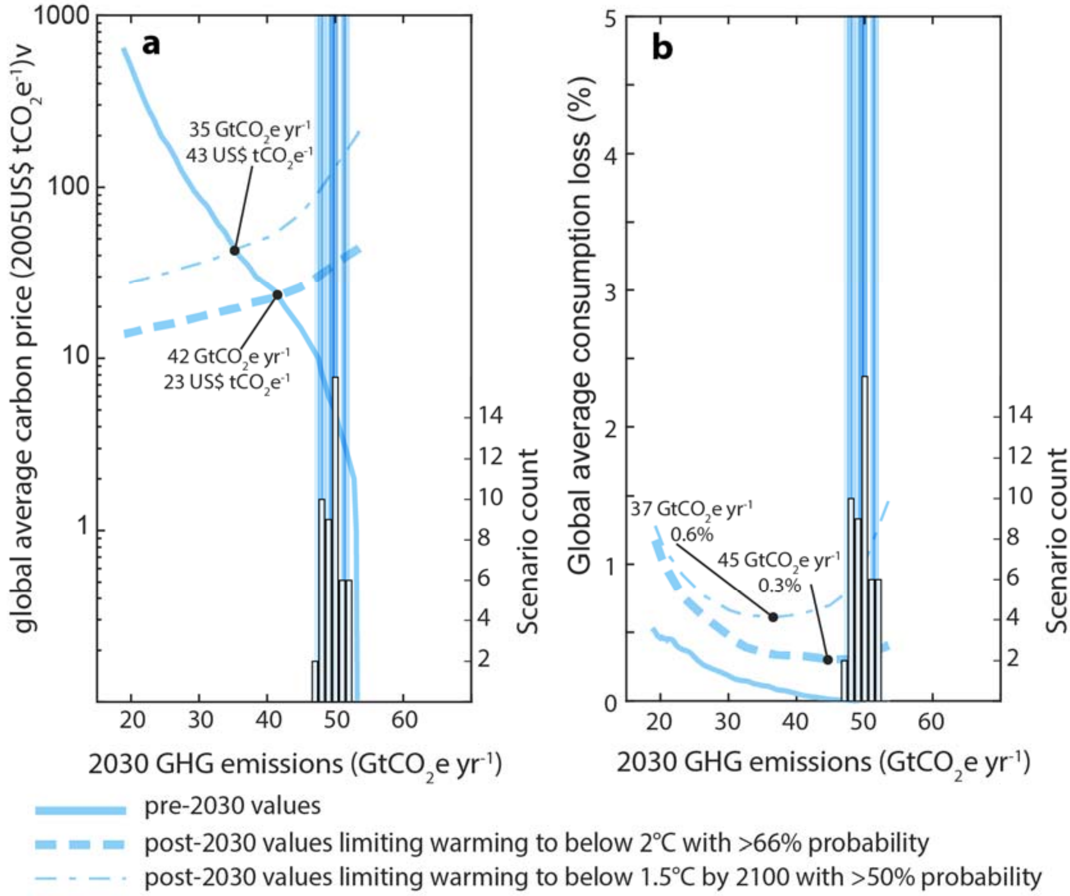

Supplementary Figure 1 | Trade-offs between 2030 NDCs and long-term temperature goals of the Paris Agreement for SSP1. Trade-offs between pre-2030 costs (solid line; global average carbon prices in panel **a**, global average consumption losses in panel **b**; see Methods for technical descriptions) and post-2030 cost in line with limiting warming to below 2°C (dashed lines) and limiting warming to below 1.5°C by 2100 (dash-dotted line) for a world with a green-growth paradigm (SSP1). The histogram and vertical lines illustrate the distribution of SSP1 NDC estimates (scenario count for histograms is shown by the right axis).

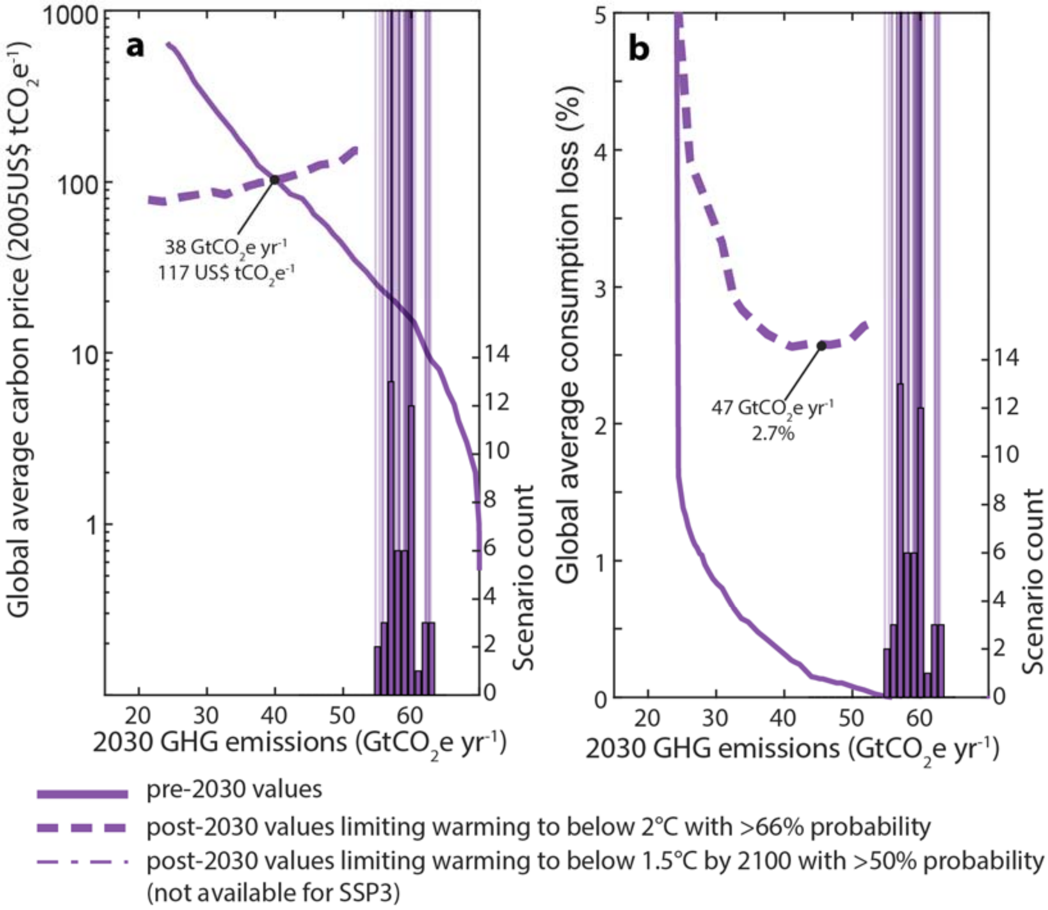

Supplementary Figure 2 | Trade-offs between 2030 NDCs and long-term temperature goals of the Paris Agreement for SSP3. Trade-offs between pre-2030 costs (solid line; global average carbon prices in panel **a**, global average consumption losses in panel **b**; see Methods for technical descriptions) and post-2030 cost in line with limiting warming to below 2°C (dashed lines) and limiting warming to below 1.5°C by 2100 (dash-dotted line) for a world characterized by regional rivalry and resurgent nationalism (SSP3). The histogram and vertical lines illustrate the distribution of SSP3 NDC estimates (scenario count for histograms is shown by the right axis). Note that in SSP3, no scenarios which limit warming to below 1.5°C with >50% probability by 2100 could be modelled.

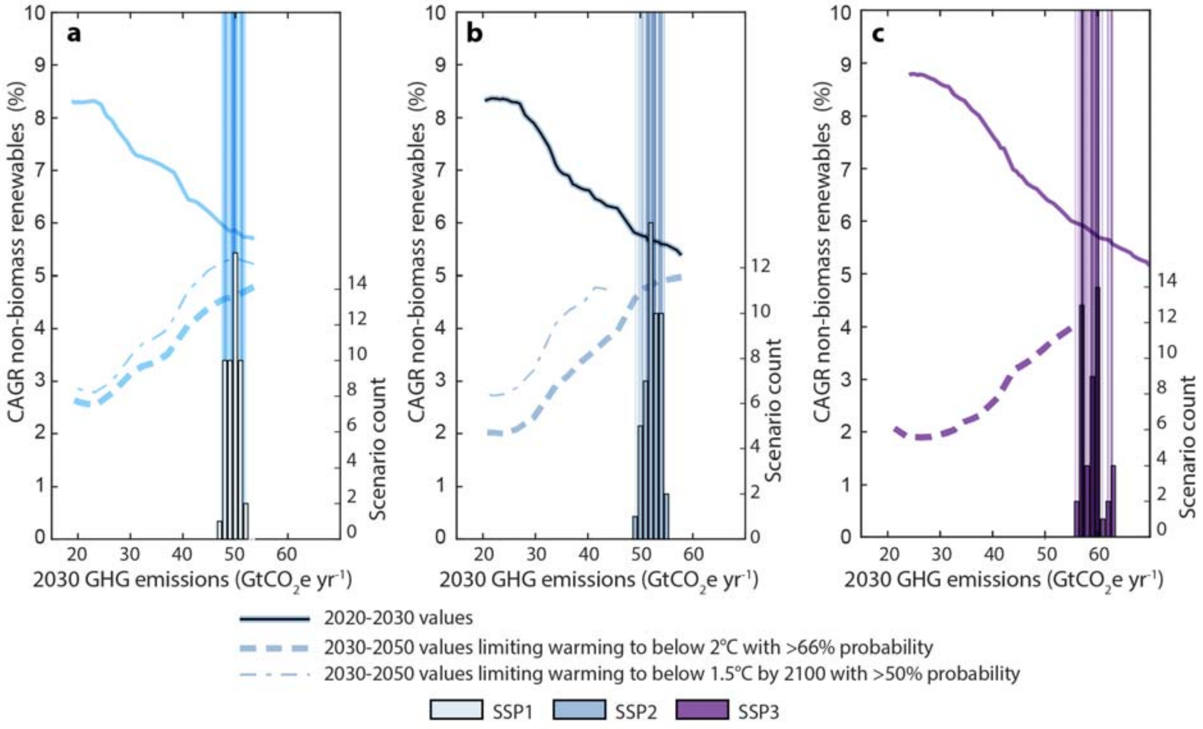

Supplementary Figure 3 | Trade-offs between 2030 NDCs and long-term temperature goals of the Paris Agreement for SSP3. Illustrative trade-offs between compound average growth rates (CAGR) of non-biomass renewable primary energy production (solar, wind, hydro, geothermal; solid line) between 2020 and 2030, and CAGR between 2030 and 2050 in line with limiting warming to below 2°C (dashed lines) and limiting warming to below 1.5°C by 2100 (dash-dotted line) for three SSP1 (SSP1, SSP2, and SSP3). The histogram and vertical lines illustrate the distribution of SSP3 NDC estimates (scenario count for histograms is shown by the right axis). Note that in SSP3, no scenarios which limit warming to below 1.5°C with >50% probability by 2100 could be modelled.

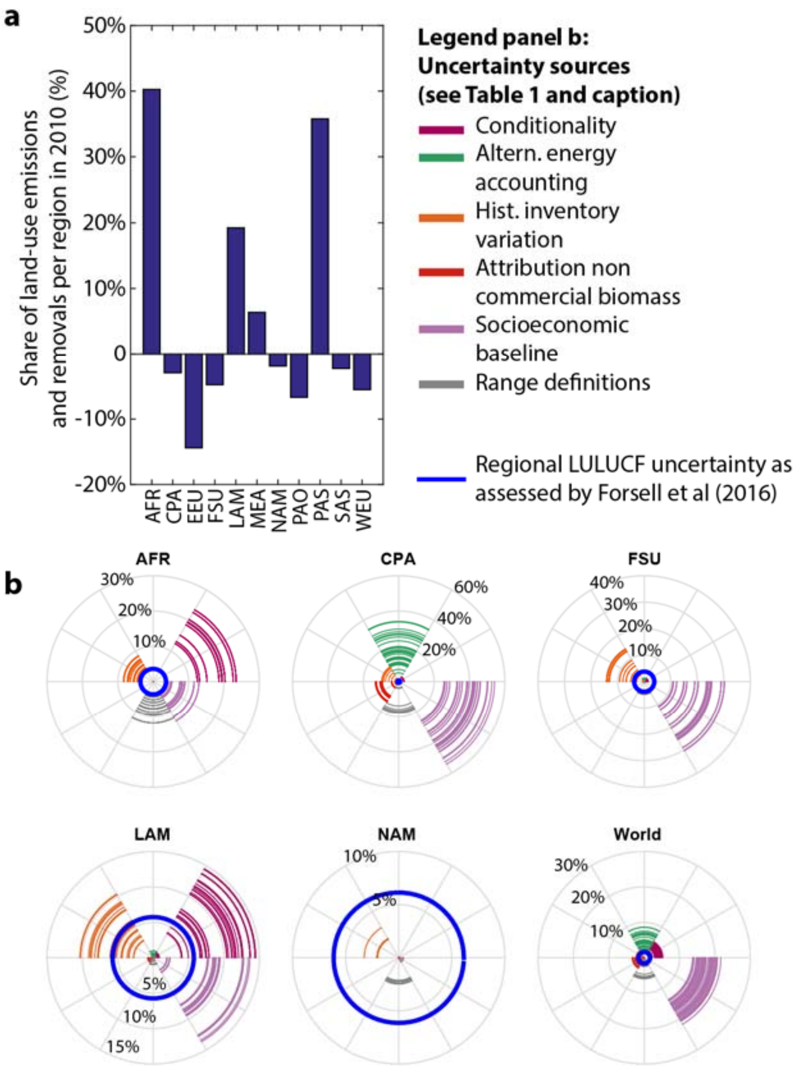

Supplementary Figure 4 | Illustration of potential influence of land-use emissions on NDC uncertainties. a, Share of year-2010 land-use emissions and removals as percentage of total regional emissions. Land-use emissions include both emissions and removals as reported in ref. 1 (fields: "land use total" and "Net emissions/removals (CO2eq)"). They are compared to the total regional GHG emissions in the MESSAGE model; **b**, estimates of the magnitude of uncertainty induced in 2030 per source relative to the median estimate, with the uncertainty in land use, land-use change, and forestry (LULUCF) contributions taken from ref. 2 and indicated by the blue circle. The blue circles show the relative magnitude of the emissions uncertainty range for single countries reported in Table 3 of ref. 2. The latter study noted that many NDCs do not contain specific targets for the LULUCF contributions. The estimates shown here thus only give a first comparison: they do not represent a full assessment of LULUCF uncertainty and they also cover only a limited set of countries. Finally, uncertainty in the LULUCF part of NDCs does not have to translate in uncertainty of the full NDC. For example, in the case of the US, the LULUCF contribution of its NDC comes with important uncertainties. However, the overall economy-wide target of its NDC is not affected by this as it applies to all sectors and is relative to a historical base year. Under the US NDC, a shortfall in mitigation in the LULUCF sector should thus be balanced by deeper reductions in other sectors.

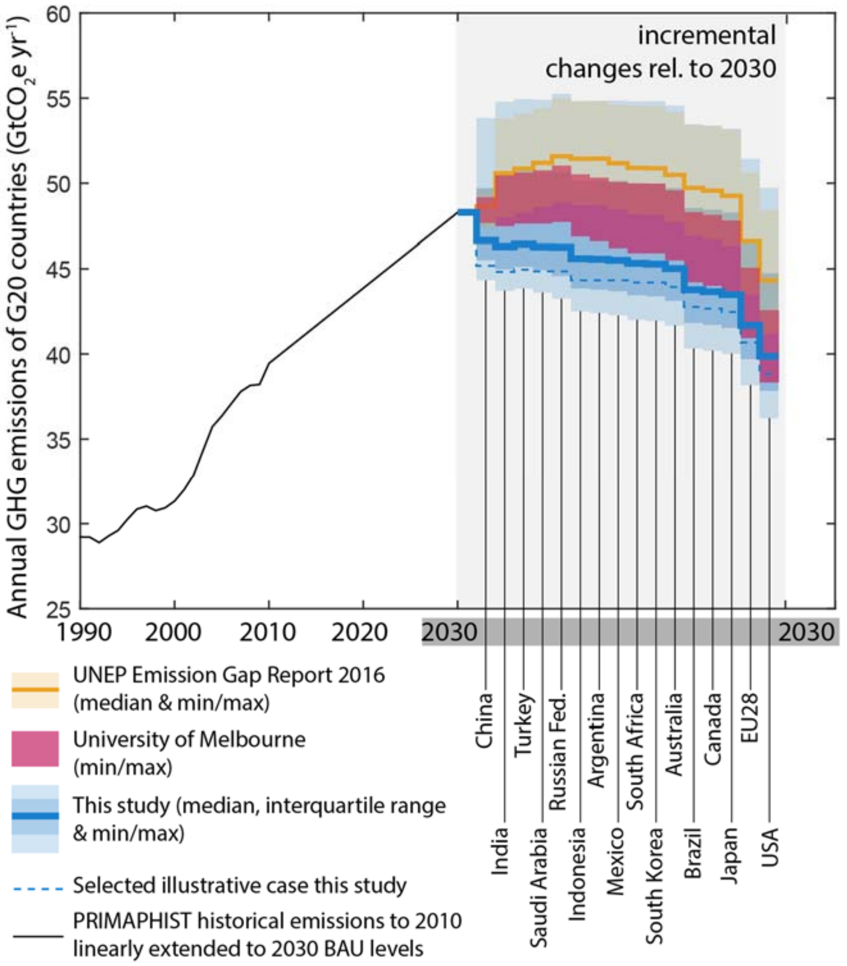

Supplementary Figure 5 | Quantification of single NDCs. Incremental changes from no-policy reference levels in 2030 in the IIASA IAM framework (blue features) compared to literature values from UNEP[3] and the University of Melbourne[4]. The 'selected illustrative case' from this study assumes an SSP2 socioeconomic development, unconditional NDCs, PRIMAPHIST historical emission inventories, direct equivalence energy accounting, and does not count non-commercial biomass towards renewable energy. The variations found in the literature fall well within our uncertainty range. Furthermore, clearly different default assumptions are applied by the assessments of the different studies. Understanding these differences will be of important in future assessments of NDCs.

Supplementary Table 1 | Estimated impact of assessed uncertainty dimensions on 2030 GHG emissions. Uncertainty ranges are minimum-maximum ranges. Emissions are expressed in GWP-100 values from ref. 5.

Global†	***AFR***	***CPA***	***EEU***	***FSU***	***LAM***	***MEA***	***NAM***	***PAO***	***PAS***	***SAS***	***WEU***
Mean emission estimate (GtCO$_2$e yr^{-1}) in 2030											
52.2	3.2	14.0	0.9	3.6	5.0	4.0	6.0	1.8	4.1	5.6	3.7
Median emission estimate (GtCO$_2$e yr^{-1}) in 2030											
51.0	3.3	13.0	0.9	3.8	5.0	3.9	6.0	1.8	4.0	5.2	3.7
Overall emission estimate incl. uncertainty‡											
45.9-61.4	2.7-4.1	10.7-20.1	0.9-1.0	3.2-4.4	4.6-5.6	3.5-4.6	5.8-6.1	1.8-1.9	3.8-4.4	4.9-6.7	3.7-3.8
Uncertainty due to socio-economic baseline variation‡											
7.0-11.1	0.1-0.4	3.4-7.1	0-0	0.4-1.1	0.1-0.7	0.6-0.7	0-0	0-0	0.1-0.2	1.6-1.7	0-0
Uncertainty due to historical emission variation‡											
0.1-1.2	0.1-0.3	0-1.2	0-0	0-0.5	0.1-0.5	0-0.2	0.1-0.2	0-0.1	0-0.1	0-0.1	0-0
Uncertainty due to conditionality of NDCs‡											
1.0-2.7	0.4-0.8	0-0.4	0-0	0-0.1	0-0.7	0.2-0.5	0-0	0-0	0.4-0.5	0-0.1	0-0
Uncertainty due to range specifications of NDCs‡											
0.2-3.0	0.1-0.4	0-2.3	0-0	0-0.2	0-0.1	0-0	0.1-0.1	0-0	0-0	0-0	0-0
Uncertainty due to alternative energy accounting methods‡											
0.1 -4.4	0-0	0.1-4.5	0-0	0-0.1	0-0.1	0-0	0-0	0-0	0-0	0-0.1	0-0
Uncertainty due to attribution of non-commercial biomass‡											
0-1.7	0-0	0-1.7	0-0	0-0	0-0	0-0	0-0	0-0	0-0	0-0	0-0

Footnotes:
† Regions are defined in Supplementary Table 2 and illustrated in Figure 3.
‡ Minimum-maximum ranges in GtCO$_2$e yr^{-1} (aggregated with GWP-100 values from ref. 5).

Supplementary Table 2 | Definition of regions in the IIASA IAM

IIASA IAM region	Definition *(list of countries)*
**AFR**	**Sub-Saharan Africa**<br>*(Angola, Benin, Botswana, British Indian Ocean Territory, Burkina Faso, Burundi, Cameroon, Cape Verde, Central African Republic, Chad, Comoros, Cote d'Ivoire, Congo, Democratic Republic of Congo, Djibouti, Equatorial Guinea, Eritrea, Ethiopia, Gabon, Gambia, Ghana, Guinea, Guinea-Bissau, Kenya, Lesotho, Liberia, Madagascar, Malawi, Mali, Mauritania, Mauritius, Mozambique, Namibia, Niger, Nigeria, Reunion, Rwanda, Sao Tome and Principe, Senegal, Seychelles, Sierra Leone, Somalia, South Africa, Saint Helena, Swaziland, Tanzania, Togo, Uganda, Zambia, Zimbabwe)*
**CPA**	**Centrally Planned Asia and China**<br>*(Cambodia, China (incl. Hong Kong), Korea (DPR), Laos (PDR), Mongolia, Viet Nam)*
**EEU**	**Central and Eastern Europe**<br>*(Albania, Bosnia and Herzegovina, Bulgaria, Croatia, Czech Republic, The former Yugoslav Rep. of Macedonia, Hungary, Poland, Romania, Slovak Republic, Slovenia, Yugoslavia, Estonia, Latvia, Lithuania)*
**FSU**	**Former Soviet Union**<br>*(Armenia, Azerbaijan, Belarus, Georgia, Kazakhstan, Kyrgyzstan, Republic of Moldova, Russian Federation, Tajikistan, Turkmenistan, Ukraine, Uzbekistan)*
**LAM**	**Latin America and the Caribbean**<br>*(Antigua and Barbuda, Argentina, Bahamas, Barbados, Belize, Bermuda, Bolivia, Brazil, Chile, Colombia, Costa Rica, Cuba, Dominica, Dominican Republic, Ecuador, El Salvador, French Guyana, Grenada, Guadeloupe, Guatemala, Guyana, Haiti, Honduras, Jamaica, Martinique, Mexico, Netherlands Antilles, Nicaragua, Panama, Paraguay, Peru, Saint Kitts and Nevis, Santa Lucia, Saint Vincent and the Grenadines, Suriname, Trinidad and Tobago, Uruguay, Venezuela)*
**MEA**	**Middle East and North Africa**<br>*(Algeria, Bahrain, Egypt (Arab Republic), Iraq, Iran (Islamic Republic), Israel, Jordan, Kuwait, Lebanon, Libya/SPLAJ, Morocco, Oman, Qatar, Saudi Arabia, Sudan, Syria (Arab Republic), Tunisia, United Arab Emirates, Yemen)*
**NAM**	**North America**<br>*(Canada, Guam, Puerto Rico, United States of America, Virgin Islands)*
**PAO**	**Pacific OECD**<br>*(Australia, Japan, New Zealand)*
**SAS**	**South Asia**<br>*(Afghanistan, Bangladesh, Bhutan, India, Maldives, Nepal, Pakistan, Sri Lanka)*
**PAS**	**Other Pacific Asia**<br>*(American Samoa, Brunei Darussalam, Fiji, French Polynesia, Gilbert-Kiribati, Indonesia, Malaysia, Myanmar, New Caledonia, Papua, New Guinea, Philippines, Republic of Korea, Singapore, Solomon Islands, Taiwan (China), Thailand, Tonga, Vanuatu, Western Samoa)*
**WEU**	**Western Europe**<br>*(Andorra, Austria, Azores, Belgium, Canary Islands, Channel Islands, Cyprus, Denmark, Faeroe Islands, Finland, France, Germany, Gibraltar, Greece, Greenland, Iceland, Ireland, Isle of Man, Italy, Liechtenstein, Luxembourg, Madeira, Malta, Monaco, Netherlands, Norway, Portugal, Spain, Sweden, Switzerland, Turkey, United Kingdom)*

Supplementary Table 3 | Overview of regional 2020 and 2030 upper limits and modelled GHG emissions in $MtCO_2e\ yr^{-1}$ for one illustrative scenario case.

Region Codes*	Region Names	No-Policy GHG emissions	Calculated GHG emission limit†,‡	Modelled GHG emissions†,‡
**2020 Targets in Mt CO_2 yr^{-1}e**				
LAM	Latin America	6,849	5,851	5,851
PAS	Other Pacific Asia	4,405	4,333	4,333
MEA	Middle East and North Africa	3,616	3,611	3,603
EEU	Eastern Europe	1,259	1,098	1,098
CPA	Centrally Planned Asia and China	13,645	15,674	12,530
NAM	North America	8,003	7,044	7,044
FSU	Former Soviet Union	3,654	4,438	3,651
WEU	Western Europe	4,958	3,995	3,995
SAS	South Asia	3,855	5,135	3,773
AFR	Sub-Saharan Africa	3,899	3,678	3,678
PAO	Pacific OECD	2,412	1,706	1,706
**2030 Targets in Mt CO_2 yr^{-1}e**				
LAM	Latin America	6,697	5,672	5,512
PAS	Other Pacific Asia	4,770	4,422	4,422
MEA	Middle East and North Africa	4,509	4,255	4,255
EEU	Eastern Europe	1,278	683	955
CPA	Centrally Planned Asia and China	14,976	16,869	12,188
NAM	North America	8,009	6,091	6,091
FSU	Former Soviet Union	3,899	4,145	3,908
WEU	Western Europe	5,177	3,381	3,796
SAS	South Asia	5,763	7,744	5,357
AFR	Sub-Saharan Africa	4,574	3,592	3,592
PAO	Pacific OECD	2,333	1,869	1,869

Footnotes:
* Regions are defined in Supplementary Table 2 and illustrated in Figure 3.
† If the modelled policy emission levels are below the calculated emission limit, this indicates that there are other more stringent constraints within that region.
‡ These illustrative values were computed for the scenario assuming a SSP2 socioeceonomic development, PRIMAPHIST historical data, unconditional NDCs, the most stringent end of range definitions, the direct equivalence energy equivalence method, and without counting non-commercial biomass energy towards renewables.

Supplementary Table 4 | Overview of regional 2020 and 2030 share [in %] comparing the calculated shares based on the Cancun pledges and national NDC targets with the actual attained shares in the policy scenario.

Region Codes	Region Names*	Constraint type	Target share‡	Modelled share‡
		**2020 Targets in %**		
LAM	Latin America	Non-Fossil Electricity Generation	7%	57%
PAS	Other Pacific Asia	Renewable Electricity Generation	7%	21%
MEA	Middle East and North Africa	Renewable Electricity Generation	4%	5%
EEU	Eastern Europe	Renewable Final Energy	18%	18%
CPA	Centrally Planned Asia and China	Non-Fossil Electricity Generation	14%	14%
NAM	North America	Renewable Electricity Generation	12%	25%
FSU	Former Soviet Union	Renewable Electricity Generation	4%	16%
WEU	Western Europe	Renewable Final Energy	18%	19%
SAS	South Asia	Renewable Electricity Generation	8%	36%
AFR	Sub-Saharan Africa	Renewable Electricity Generation	7%	35%
PAO	Pacific OECD	Renewable Primary Energy	8%	15%
		**2030 Targets in %**		
LAM	Latin America	Renewable Primary Energy	17%	24%
PAS	Other Pacific Asia	Non-Fossil Primary Energy	5%	19%
MEA	Middle East and North Africa	Renewable Electricity Generation	1%	6%
CPA	Centrally Planned Asia and China	Non-Fossil Primary Energy	21%	23%
SAS	South Asia	Non-Fossil Electricity Generation	34%	42%
AFR	Sub-Saharan Africa	Renewable Primary Energy	0%	48%

Footnotes:
* Regions are defined in Supplementary Table 2 and illustrated in Figure 3.
‡ These illustrative values were computed for the scenario assuming a SSP2 socioeceonomic development, PRIMAPHIST historical data, unconditional NDCs, the most stringent end of range definitions, the direct equivalence energy equivalence method, and without counting non-commercial biomass energy towards renewables.

Supplementary Table 5 | Overview of regional 2020 and 2030 total installed capacity [in GW] comparing the calculated capacity based on the national targets and the actual installed capacity in the modelled policy scenario.

Region Codes	Region Names*	Constraint type	Target capacity‡	Modelled capacity‡
		**2020 Targets in GW**		
LAM	Latin America	Bioenergy	9	10
LAM	Latin America	Hydro	124	214
PAS	Other Pacific Asia	Wind	16	53
CPA	Centrally Planned Asia and China	Solar	10	10
CPA	Centrally Planned Asia and China	Nuclear	80	83
CPA	Centrally Planned Asia and China	Wind	200	242
CPA	Centrally Planned Asia and China	Hydro	270	270
WEU	Western Europe	Nuclear	10	143
WEU	Western Europe	Wind	20	269
SAS	South Asia	Solar	20	19
SAS	South Asia	Bioenergy	7	39
SAS	South Asia	Wind	40	50
SAS	South Asia	Hydro	8	53
PAO	Pacific OECD	Solar	34	33
PAO	Pacific OECD	Wind	38	89
		**2030 Targets in GW**		
SAS	South Asia	Solar	102	97
SAS	South Asia	Nuclear	63	61
SAS	South Asia	Bioenergy	10.2	36
SAS	South Asia	Wind	60	101
SAS	South Asia	Hydro	12.1	57
CPA	Centrally Planned Asia and China	Solar	100	100
CPA	Centrally Planned Asia and China	Wind	200	564
AFR	Sub-Saharan Africa	Solar	16	14
AFR	Sub-Saharan Africa	Wind	0	51
AFR	Sub-Saharan Africa	Hydro	1	42
PAO	Pacific OECD	Bioenergy	1	2
PAO	Pacific OECD	Solar	2	32
PAO	Pacific OECD	Nuclear	27	49
PAO	Pacific OECD	Wind	0	98
PAO	Pacific OECD	Hydro	2	54
PAO	Pacific OECD	Geothermal	0.3	0.4

Footnotes:
* Regions are defined in Supplementary Table 2 and illustrated in Figure 3.
‡ These illustrative values were computed for the scenario assuming a SSP2 socioeceonomic development, PRIMAPHIST historical data, unconditional NDCs, the most stringent end of range definitions, the direct equivalence energy equivalence method, and without counting non-commercial biomass energy towards renewables.

SUPPLEMENTARY REFERENCES

1. FAOSTAT. FAOSTAT Emissions Database. February 8, 2016 ed. Rome: Food and Agricultural Organization of the United Nations; 2016.
2. Forsell N, Turkovska O, Gusti M, Obersteiner M, Elzen Md, Havlik P. Assessing the INDCs' land use, land use change, and forest emission projections. *Carbon Balance and Management* 2016, **11**(1)**:** 26.
3. UNEP. The Emissions Gap Report 2016. Nairobi, Kenya: UNEP; 2016 November 2016.
4. Meinshausen M. INDC Factsheets. Melbourne, Australia: Australian-German Climate and Energy College / University of Melbourne; 2015.
5. IPCC. Climate Change 1995: The Science of Climate Change. Contribution of WGI to the Second Assessment Report of the Intergovernmental Panel on Climate Change. Cambridge, UK: Cambridge University Press; 1996.
